# Development of a real-time PCR assay and comparison to CHROMagar^TM^ STEC to screen for Shiga toxin-producing *Escherichia coli* in stool, Cape Town, South Africa

**DOI:** 10.4102/ajlm.v6i1.609

**Published:** 2017-12-14

**Authors:** John B. Kalule, Karen H. Keddy, Anthony Smith, Mark P. Nicol, Lourens Robberts

**Affiliations:** 1 Faculty of Health Sciences, University of Cape Town, Cape Town, South Africa; 2 Center for Enteric Disease Research Unit, National Institute for Communicable Diseases, Johannesburg, South Africa

## Abstract

**Introduction:**

Shiga toxin-producing *Escherichia coli* (STEC) is an emerging infectious pathogen which could lead to haemolytic uremic syndrome. Even though previous studies have compared the performance of CHROMagar^TM^STEC to real-time polymerase chain reaction (PCR) in Europe, no study has been done to assess its performance on African isolates.

**Objectives:**

This project aimed to validate and test an in-house-developed duplex real-time PCR and use it as a reference standard to determine the performance of CHROMagar^TM^STEC on African isolates from diarrhoeic stool samples.

**Methods:**

This study evaluated STEC diagnostic technology on African isolates. An in-house-developed duplex real-time PCR assay for detection of *stx*_*1*_ and *stx*_*2*_ was validated and tested on diarrhoeic stool samples and then used as a reference standard to assess the performance of CHROMagar^TM^STEC. Real-time PCR was used to screen for *stx* in tryptic soy broth and the suspected STEC isolates, while conventional PCR was used to detect the other virulence genes possessed by the isolates.

**Results:**

The real-time PCR limit of detection was 5.3 target copies/μL of broth. The mean melting temperature on melt-curve analysis for detection of *stx*_*1*_ was 58.2 °C and for *stx*_*2*_ was 65.3 °C. Of 226 specimens screened, real-time PCR detected *stx* in 14 specimens (6.2%, 95% confidence interval = 3.43% – 10.18%). The sensitivity, specificity, negative predictive value and positive predictive value of the CHROMagar^TM^STEC were 33.3%, 77.4%, 95.3% and 11.3%.

**Conclusions:**

The in-house developed real-time PCR assay is a sensitive and specific option for laboratory detection of STEC as compared to CHROMagar^TM^STEC in this setting.

## Introduction

Globally, food- and water-borne outbreaks of both O157 and non-O157 Shiga toxin-producing *Escherichia coli* (STEC) have been successfully detected due to the availability of good baseline data and effective active laboratory-based surveillance systems.^1,2,3,4^ Early detection of outbreaks is important to minimise morbidity, mortality and associated economic losses.^5^ There is a lack of good baseline data on STEC in Africa, which can be attributed to a lack of laboratory resources and the surveillance strategy employed. STEC has been implicated in outbreaks of bloody diarrhoea in sub-Saharan countries;^6,7,8^ however, these have been difficult to track and manage due to laboratory weakness.^9,10^ Furthermore, typical haemolytic uremic syndrome, which is overwhelmingly caused by STEC, was reported as the leading contributor to acute renal failure in paediatric patients at a South African academic hospital.^11^ Even though several studies have evaluated the performance of CHROMagar^TM^STEC by comparison to molecular and antigen detection methods in developed countries,^12,13^ no study has so far evaluated its performance in Africa. This is necessary, especially given that there are geographical differences in characteristics of STEC that are dependent on the index of suspicion for the different STEC serotypes and on the availability of suitable laboratory methods to detect them.^14^

In many South African (and African) laboratories, stool specimens are not routinely tested for STEC, although physicians may request tests specific for *E. coli* serotype O157:H7, if it is clinically suspected. Testing is based on the non-sorbitol fermenting property using sorbitol MacConkey and only on request by a physician. This practice is of concern, is misleading and underestimates the real magnitude of STEC, since not all serotype O157 strains are non-sorbitol fermenting (O157: NM), and over 470 non-O157 serotypes have been attributed to clinical disease.^15^

Laboratory capacity for molecular detection is increasingly available in African countries and may, in some cases, be simpler than culture-based detection. This project, therefore, aimed to validate and test diarrhoeic stool samples by using an in-house developed duplex real-time polymerase chain reaction (PCR) and use it as a reference standard to determine the performance of CHROMagar^TM^STEC on African isolates. The duplex assay was used to screen tryptic soy broth (TSB) for *stx* following overnight stool enrichment, and conventional PCR was used to screen for the other diarrhoeagenic *E. coli* virulence genes. Diarrhoeagenic *E. coli* were serotyped, and *stx*-positive isolates were tested for Shiga toxin production using immunochromatography.

## Methods

### Ethical approval

This study was approved by the ethics and research committee of the Faculty of Health Sciences at the University of Cape Town (HREC REF: 014/2015).

### Study design

**FIGURE 1:**
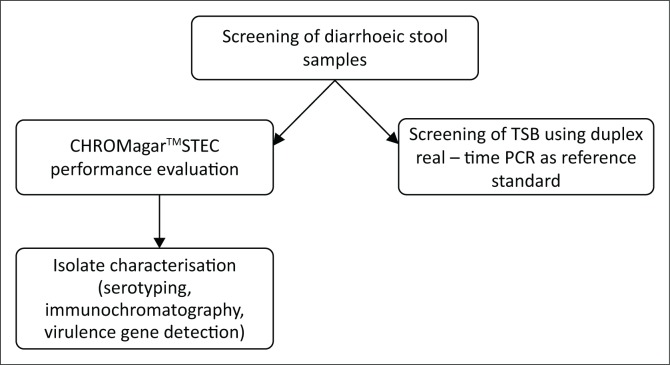
Summary of methods used in this study.

This study validated an in-house-developed duplex real-time PCR assay for detection of *stx*_*1*_ and *stx*_*2*_. The assay was then tested on diarrhoeic stool samples at a tertiary referral hospital and was used as a reference standard to assess the performance of a commercial chromogenic medium (CHROMagar^TM^STEC) for STEC screening (Figure 1).

### Target plasmid preparation

The real-time PCR previously described by Grys et al.^16^ was used to amplify *stx*_*1*_ and *stx*_*2*_ gene targets from a STEC O157:H7 NCTC control strain (C4193-1) with both *stx*_*1*_ (subtype 1a) and *stx*_*2*_ (subtype 2a). PCR amplicon size was confirmed visually by agarose gel detection (~208 bp for *stx*_*1*_ and ~204 bp for *stx*_*2*_) before confirmation by sequencing using the Big Dye® Terminator v3.1 Cycle Sequencing Kit (Life Technologies Corporation, Carlsbad, California, United States). We used primers 1a and 2a (Table 1) for unidirectional Sanger sequencing of the amplicons. Resultant sequences were then trimmed and submitted for BLAST analysis against the NCBI database and confirming *stx*_*1*_ or *stx*_*2*_ target sequences in comparison to O157:H7 EDL933 (NCBI Reference: NC_002655.2).^17^ Purified amplicons (Mini Elute Gel extraction kit, Qiagen, Madrid, Spain) were cloned using CloneJet PCR cloning kit (Thermofisher Scientific, Austin, Texas, United States) into a pJet 1.2/blunt vector using the sticky end cloning protocol and transfected into the JM109 competent cells by calcium chloride transformation. Plasmids containing *stx*_*1*_ and *stx*_*2*_ were separately extracted using a Genopure plasmid Maxi kit (Roche Life Sciences, Rotkreuz, Switzerland) and quantified by spectrophotometry. To verify successful preparation purified plasmids were subjected to PCR amplification using primers 1a and 1b for *stx*_*1*_ and 2a and 2b for *stx*_*2*_ with amplicon size visually confirmed by agarose gel detection and subsequent sequence analysis. Plasmid quantification was determined spectrophotometrically employing the BioDrop-μLite (Isogen Life Science, B.V, Veldzigt, Netherlands). The A_260_ was used to calculate the plasmid concentration expressed as the number of molecules/μL.

**TABLE 1:** Primers and probes used for real-time polymerase chain reaction.

Primers/probes	5^!^	Sequence	3^!^	Reference
*stx*_*1*_a-primer		CAAGAGCGATGTTACGGT		(16)
*stx*_*1*_b-primer		AATTCTTCCTACACGAACAGA		(16)
*stx*_*1*_f-probe		CTGGGGAAGGTTGAGTAGCG	Fluorescein	(16)
*stx*_*1*_r-probe	CAL Fluor 610	CCTGCCTGACTATCATGGACA	3′ phosphor	(16)
*stx*_*2*_a-primer		GGGACCACATCGGTGT		(16)
*stx*_*2*_b-primer		CGGGCACTGATATATGTGTAA		(16)
*stx*_*2*_f-probe		CTGTGGATATACGAGGGCTTGATGTC	Fluorescein	(16)
*stx*_*2*_r-probe	CAL Fluor 610	ATCAGGCGCGTTTTGACCATCT	3′ phosphor	(16)

### Polymerase chain reaction assay validation

To assess the potential for PCR cross-reactivity and assess the analytical specificity of the hybridisation probe-based real-time PCR described by Grys et al.,^16^ the primer and probe sequences were subjected to BLAST analysis on the NCBI database. The PCR reaction was optimised for use on the LightCycler^®^480 Instrument II (Roche Life Sciences, Rotkreuz, Switzerland) employing the LightCycler^®^ 480 Probes Master mastermix (Roche Life Sciences, Rotkreuz, Switzerland) with modification to the thermal cycling conditions for amplification consisting of denaturation at 95 °C for 10 min followed by 45 cycles of 95 °C for 5 s, 56 °C for 5 s and 72 °C for 15 s. A positive amplification signal was defined as an increase in fluorescence signal that crossed the threshold before 30 cycles. Amplicon identity was determined using the melt-curve analysis program of 95 °C for 30 s, 40 °C for 60 s and 85 °C for 5 s with continuous fluorescence acquisition. The Multi-color HybProbe detection format was used for analysis, combining the Red 610, Red 640 and FAM filter pairs (LightCycler^®^480 Instrument II Manual, Roche Life Sciences, Rotkreuz, Switzerland). The resulting amplicon size was visualised using agarose gel electrophoresis and subjected to DNA sequencing and BLAST alignment to reference *stx*_*1*_*a* and *stx*_*2*_*a* sequences (NC_002655.2). To mimic the sample matrix for sensitivity determination, TSB was inoculated with a pea-size amount of stool (from a single donor) shown to be *stx*-negative by PCR. To this inoculated broth 1 mL of plasmid stock (5.3*10^6^ copies/μL) containing both *stx*_*1*_ and *stx*_*2*_ was added and serially diluted eight times in 9 mL of TSB, to a lowest dilution of 1:10^8^ (53 plasmid copies/ml). Nucleic acid extraction was performed on 200 μL broth employing the MagNApure LC instrument (Roche Diagnostics, Rotkreuz, Switzerland) to yield 100 μL of extract. Initially, real-time PCR was performed in triplicate using a template from each of the eight dilutions to estimate a limit of detection (LOD). Subsequently, real-time PCR was performed in eight replicates on the dilution with the estimated LOD, as well as one dilution above and one dilution below the estimate. The LOD was defined as the lowest plasmid concentration spiked into TSB, before nucleic acid extraction, yielding a positive signal, as described above in all eight replicates. Nucleic acid extractions from STEC subtypes 1d (Reference strain MH1813, GenBank accession No. AY170851), 2b (Reference strain EH250, GenBank accession No. AF043627), 2c (Reference strain 031, GenBank accession No. L11079), 2d (Reference strain C165-02, GenBank accession No. DQ059012), 2e (Reference strain S1191, GenBank accession No. M21534), 2f (Reference strain T4/97, GenBank accession No. AJ010730) and 2g (Reference strain 7V, GenBank accession No. AY286000) were also subjected to PCR amplification to assess impact of strain variation on detection. The reproducibility of melting temperature assessment for *stx*_*1*_ and *stx*_*2*_ differentiation was determined by testing 24 replicates of TSB spiked with cloned *stx*_*1*_ and *stx*_*2*_ plasmids. To further assess the reproducibility of melting temperature across the subtypes, three *stx*_*1*_ subtypes and seven *stx*_*2*_ subtypes were tested similarly.

### Clinical specimen testing

Between September 2014 and May 2015, we collected same day residual stool after routine testing from 226 consecutive stool specimens (the stool samples were transported in a temperature regulated box and processed within 12 h of collection) from the National Health Laboratory Services located at the Groote Schuur Hospital in Cape Town, South Africa, a tertiary care academic teaching laboratory affiliated with the University of Cape Town. This tertiary academic hospital serves the greater Cape Town area.

A pea-sized stool sample was inoculated in 90 mL of TSB and vortexed before incubation at 37 °C for 18 h. Two hundred microlitres of broth were subsequently extracted employing the MagNA Pure LC Total Nucleic Acid isolation kit (Roche Diagnostics, Rotkreuz, Switzerland) using the total variable elution volume protocol and following the manufacturer’s manual (version 14) to yield 100 μL of nucleic acid extract.

In addition, CHROMagar^TM^STEC (CHROMagar Microbiology, Paris, France) was inoculated with a loop full of overnight inoculated broth and incubated at 37 °C for 18 h. Bright mauve colonies (up to five mauve colonies were picked per sample, depending on the number of mauve colonies formed) were sub-cultured onto MacConkey agar with crystal violet, sorbitol MacConkey agar, and 2% blood agar (Green point Media, National Health Laboratory Service, Albertynshof, South Africa). *E. coli* was presumptively identified as lactose-positive, oxidase-negative, spot indole-positive and pyrrolidonyl arylamidase (PYR)-negative with confirmatory identification using VITEK 2 (bioMerieux, Inc., Durham, North Carolina, United States).

### Isolate characterisation

Isolates yielding mauve colonies on CHROMagar^TM^STEC and presumptively identified as *E. coli* were subjected to *stx* characterisation employing the real-time PCR assay characterised herein. Other diarrhoeic *E. coli* virulence genes, including the fimbrial adhesion gene for diffusely adherent *E. coli,* the anti-aggregation protein transporter gene for enteroaggregative *E. coli*, heat-stable and heat-labile enterotoxin genes of enterotoxigenic *E. coli*, the intimin coding gene *eae* for enteropathogenic *E. coli* (EPEC) and the bundle-forming pili gene for the typical EPEC, were determined using standard gel-based PCR as previously described using primers as shown in Table 2.^18^

**TABLE 2:** Primers used for detection of virulence genes by conventional polymerase chain reaction.

	Genes	Primers	Primer sequence	Product size
A	*eae*	*eae*-F	TCAATGCAGTTCCGTTATCAGTT	482 bp
		*eae*-R	GTAAAGTCCGTTACCCCAACCTG	
	*bfp*	*bfp*-F	GGAAGTCAAATTCATGGGGGTAT	298 bp
		*bfp*-R	GGAATCAGACGCAGACTGGTAGT	
	*stx*_*1*_	*stx*_*1*_-F	CAGTTAATGTGGTGGCGAAGG	348 bp
		*stx*_*1*_-R	CACCAGACAATGTAACCGCTG	
	*stx*_*2*_	*stx*_*2*_-F	ATCCTATTCCCGGGAGTTTACG	584 bp
		*stx*_*2*_-R	GCGTCATCGTATACACAGGAGC	
B	*Est*	*ST*-F	ATTTTTCTTTCTGTATTGTCTT	190 bp
		*ST*-R	CACCCGGTACAAGCAGGATT	
	*Elt*	*LT*-F	GGCGACAGATTATACCGTGC	440 bp
		*LT*-R	CGGTCTCTATATTCCCTGTT	
C	*ipa*	*ipaH*-F	CTCGGCACGTTTTAATAGTCTGG	933 bp
		*ipaH*-R	GTGGAGAGCTGAAGTTTCTCTGC	
	*aat*	pCVD432-F	CTGGCGAAAGACTGTATCAT	630 bp
		pCVD432-R	CAATGTATAGAAATCCGCTGTT	
	*DaaC*	*daaC*-F	CAGGTCATCCGGTCAGTCGG	212 bp
		*daaC*-R	CAATGCCACGTACAACCGGC	

ST, heat-stable; LT, heat-labile.

To confirm Shiga toxin production among *stx*-positive isolates, the Immunocard STAT!^®^ EHEC (Meridian Biosciences, Inc., Cincinnati, Ohio, United States) was used to detect Shiga toxin 1 and 2 (by employing immunochromatography with toxin-directed monoclonal antibodies labelled with red-coloured gold particles). All mauve isolates found to carry virulence genes were serotyped at the Centre for Enteric Diseases, National Institute of Communicable Disease, Johannesburg, by employing antisera (Statens Serum Institut, Copenhagen, Denmark) and the detection of somatic O-antigens as previously described.^19,20^ H-antigen serotyping was not undertaken.

### Statistical analysis

Data on the possession of virulence genes, cultural characteristics on different media, serotypes and Shiga toxin production was entered in Microsoft Office Excel 2010 (Microsoft Corp.,Redmond, Washington, United States) and then exported to EPIINFO^TM^ 7.1.5.2 (Centers for Disease Control and Prevention, Atlanta, Georgia, United States) for analysis. Using the LightCycler 480 II software, efficiency of the in-house real-time PCR assay was determined. The amplification curves and the melting peaks were used to differentiate between *stx*_*1*_ and *stx*_*2*_.

## Results

### Real-time polymerase chain reaction validation

**FIGURE 2:**
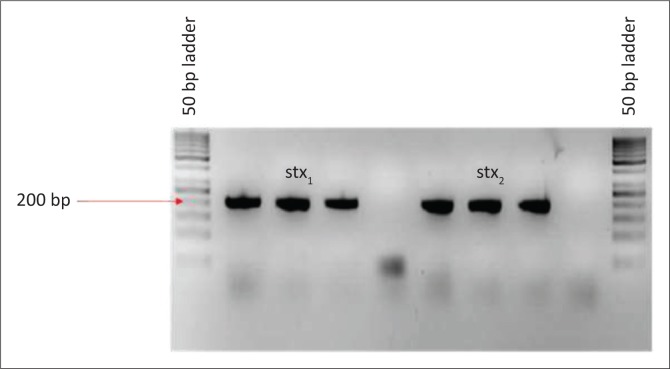
Polymerase chain reaction amplicons of *stx*_1_ and *stx*_2_ after cloning.

**FIGURE 3:**
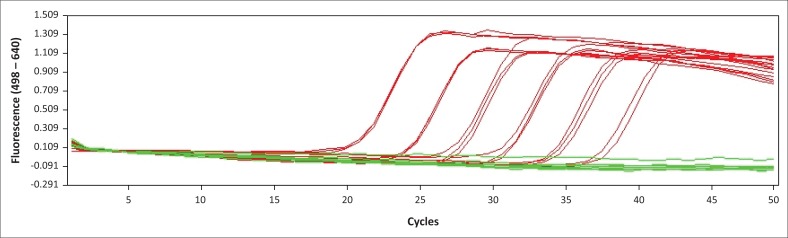
Amplification curves for the validated duplex real-time polymerase chain reaction for detection of the *stx* gene

**FIGURE 4:**
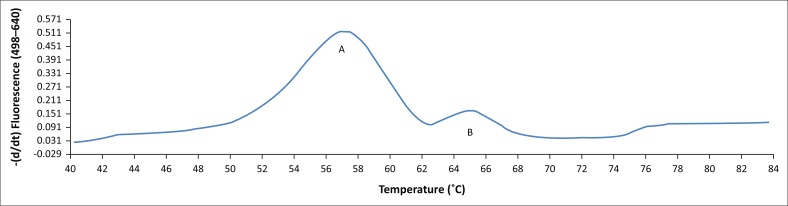
Melting peaks for *stx*_*1*_ – peak A (subtypes other than 1d – 55.7 °C) and *stx*_*2*_ – peak B (65.3 °C) detected in the same run.

**FIGURE 5:**
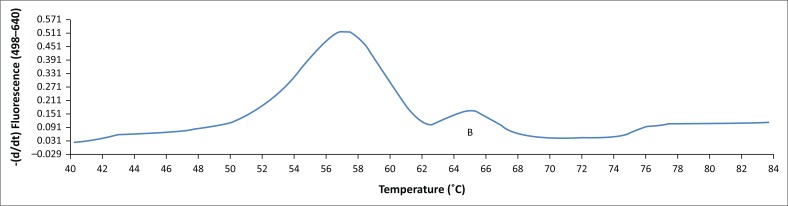
Melting peaks for optimised duplex real-time polymerase chain reaction showing *stx*_*2*_ peak B (65.3°C).

**FIGURE 6:**
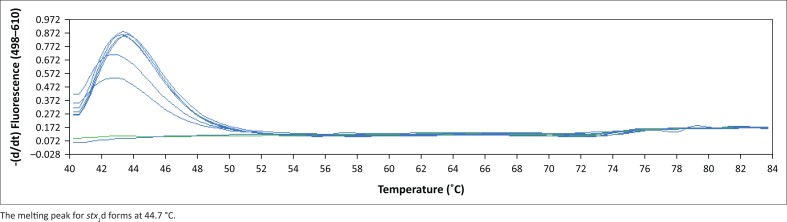
Melting peaks for the detection of *stx*_*1*_d using the duplex real-time polymerase chain reaction assay.

**FIGURE 7:**
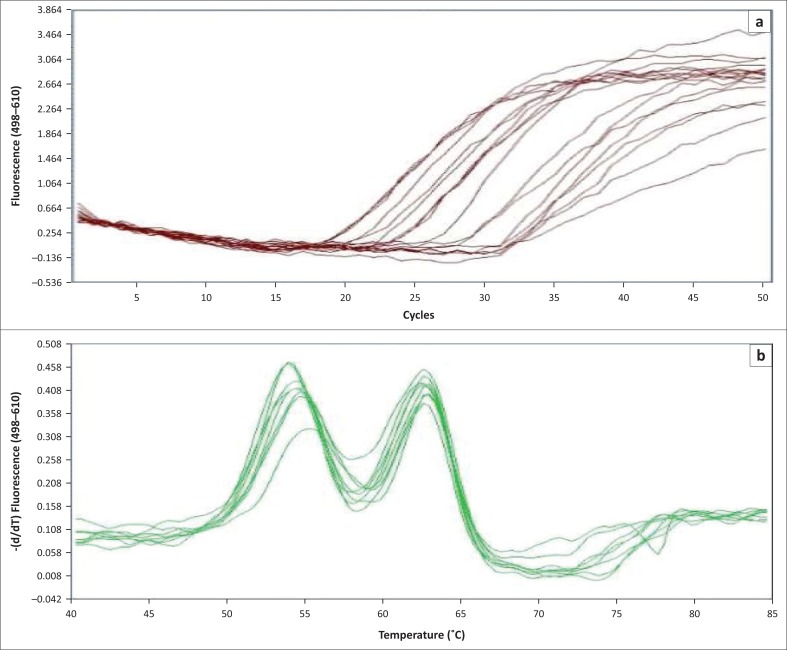
Amplification curves and melting peaks obtained on screening tryptic soy broth using the in-house optimised real-time polymerase chain reaction assay. (A) Amplification curves. (B) Melting peaks for the duplex real-time polymerase chain reaction assay.

The BLAST analysis of the primers and probe sequence specificity yielded no significant homology to non-*stx* targets (data not shown). Real-time PCR amplicons generated were confirmed as 208 bp for *stx*_*1*_ and 204 bp for *stx*_*2*_ (Figure 2). Sequencing and BLAST analysis confirmed the identity of both *stx*_*1*_ and *stx*_*2*_ amplicons. The serially diluted plasmid-stool-TSB was successfully amplified in 8/8 replicates in the sixth dilution, whereas the seventh dilution yielded an amplification signal in 3/8 replicates, yielding a LOD of 5.3 target copies/μL of broth. All other *stx* subtypes investigated (s*tx*_*1*_a, *stx*_*1*_b, *stx*_*2*_a, *stx*_*2*_b, *stx*_*2*_c, *stx*_*2*_d, *stx*_*2*_e, *stx*_*2*_f, and *stx*_*2*_g) were successfully amplified by this assay (data not shown). *stx*_1_ and *stx*_2_ were successfully distinguished by a melting temperature of 58.2 °C (SD = 0.033) and 65.3 °C (SD = 0.037) (Figures 3–6). The T_m_ for *stx*_*2*_ subtypes 2a, 2b, 2c, 2d, 2e, 2f and 2g were the same at 65.3 °C (SD = 0.037, 0.041, 0.035, 0.039, 0.034, 0.033 and 0.032, respectively), whereas that of 1d was 44.7 °C (SD = 0.042). The efficiency of the assay was 1.99 as calculated from the amplification curves generated using the Light Cycler^®^ 480 software. The duplex assay detected both targets in the same run, and these could be differentiated by the melt curve with two distinct peaks at 58.2 °C for *stx*_*1*_ and 65.3 °C for *stx*_*2*_ (Figure 7).

### Clinical specimens

**FIGURE 8:**
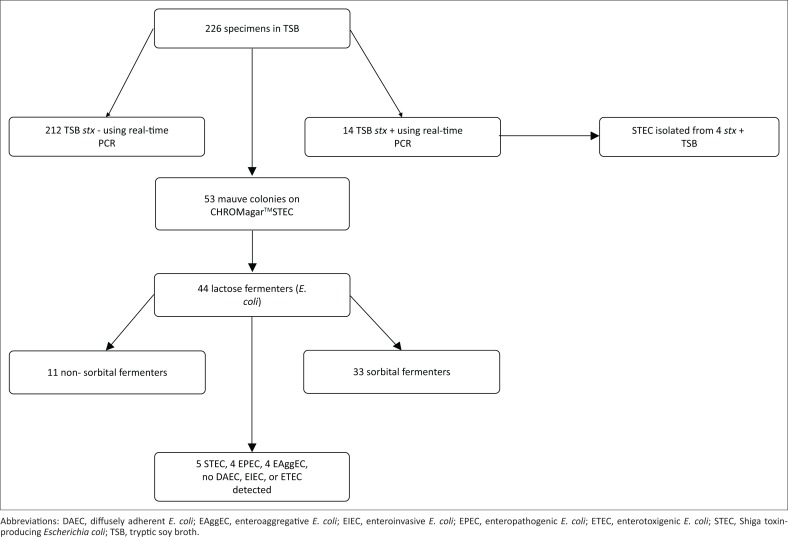
Summary of isolate characterisation results.

Of the 226 specimens screened, real-time PCR detected Shiga toxin genes in 14 samples (6.2%), comprising 8 *stx*_*1*_, 5 *stx*_*2*_ and 1 specimen containing both *stx*_*1*_ and *stx*_*2*_. CHROMagar^TM^STEC yielded mauve colonies from 23.45% (53/226) of the stool broth cultures (Figure 8).

#### Performance of CHROMagar^TM^STEC

Of the 53 mauve isolates, 48 were negative for *stx* genes using the validated real-time PCR assay. Of the 14 broths that were positive on PCR, nine did not yield any mauve colonies on CHROMagar^TM^STEC culture.

#### Isolate characteristics

Forty-four (83%) of the 53 mauve colonies fermented lactose on MacConkey agar with crystal violet. Eleven (25%) of the 44 lactose fermenters were non-sorbitol-fermenting. Real-time PCR on the 44 *E. coli* confirmed the presence of *stx* genes in five (11%), whereas 39 were negative for the *stx* gene. Real-time PCR was not done on the nine non-lactose fermenting isolates as these were found not to be *E. coli* on biochemical testing. Four of the five *stx* positive *E. coli* colonies were also positive in the real-time PCR broth assay. Of the 39 *stx*-negative *E. coli*, only four (12.5%) carried *eae* genes, whereas four possessed *aat* genes. Of the four *eae* positive isolates, two also had the *bfp* genes and were typical EPEC. The other two *eae* positive isolates did not possess the *bfp* genes and were classified as atypical EPEC. The four enteroaggregative *stx*-negative isolates all belonged to *E. coli* serotype O104. All the typical EPEC belonged to serotype O55, whereas one of the two atypical EPEC belonged to serotype O101. The atypical EPEC serotype O101 was from *stx*_*2*_ positive broth. The other atypical EPEC isolate was untypeable. No diffusely adherent *E. coli*, enteroinvasive *E. coli* or enterotoxigenic *E. coli* were detected. None of the 53 *E. coli* isolates that were screened by immunochromatography was positive for Shiga toxins. For the CHROMagar^TM^STEC, sensitivity was 33.3%, specificity was 77.4%, negative predictive value was 95% and positive predictive value was 9.4% (Table 3).

**TABLE 3:** Performance of CHROMagar^TM^STEC compared with real-time polymerase chain reaction assay.

	In-house developed assay			
**CHROMagar**TM**STEC**		+	_	Total
	+	5	48	53
	_	9	164	173
**Total**		**14**	**212**	**226**

CHROMagar^TM^STEC sensitivity = 5/14*100 = 33.3%

CHROMagar^TM^STEC specificity = 164/212*100 = 77.4%

CHROMagar^TM^STEC positive predictive value = 5/53*100 = 9.4%

CHROMagar^TM^STEC negative predictive value = 164/173*100 = 95%

## Discussion

We validated the use of a previously described duplex real-time PCR assay with modification able to detect and differentiate *stx*_*1*_ (melting temperature = 58.2 °C) and *stx*_*2*_ (melting temperature = 65.3 °C) from overnight broth enrichment with a LOD of 5.3 target copies/μL broth. This assay was able to detect both *stx*_*1*_ and *stx*_*2*_ in the same run, thus potentially reducing process turn-around time in a busy laboratory setting. Timely reporting of STEC infections is important, because use of certain antibiotics is contraindicated in STEC infections.

Compared to the validated duplex real-time PCR, CHROMagar^TM^STEC showed a sensitivity of 33%, specificity of 77.4%, negative predictive value of 9.4% and positive predictive value of 95% for detection of STEC in stool following TSB enrichment. Of the 53 mauve isolates, 48 were negative for *stx* genes on use of the validated real-time PCR, whereas nine of the 14 PCR-positive broths did not yield any mauve colonies when cultured on CHROMagar^TM^STEC. Reasons for the poor performance of this medium in relation to the in-house developed duplex real-time PCR include the following: (1) delays in reporting of diarrhoea cases to a tertiary hospital (where samples were collected) may have led to loss of *stx* genes; STEC numbers are sharply reduced in stool after one week of illness, and the Shiga toxin genes might be lost by the bacteria.^21^ (2) CHROMagar^TM^STEC selects for tellurite resistant strains but misses the tellurite susceptible STEC whose prevalence in this setting is not known.

For a chromogenic medium to be considered for routine screening purposes, it must have high specificity so as not to waste scarce laboratory resources on false positives. The false positivity rate in this setting (48/53 [90.6%]) is higher than has previously been reported in Europe (16.3% reported by Gouali et al., 2013 and 18.3% by Wylie et al., 2013).^12,22^

Similar studies to evaluate this medium were done in Canada, Finland, and Germany, all of which reported high sensitivities for STEC serotypes O26, O111, O121, O145, O118, and O157.^13,22,23^ The specificity of 77.4% noted in this study was low compared to values (between 95.8% and 98.9%) reported in similar studies done in Europe. The difference in sensitivity and specificity could be explained by the differences in the patient characteristics (whether they present with haemolytic uremic syndrome and or bloody diarrhoea or not). Unlike the studies in Europe, this study did not focus on only patients with haemolytic uremic syndrome or bloody diarrhoea. Additionally, the distribution of tellurite resistant STEC (which is targeted by CHROMagar^TM^STEC) in this setting is not known.

The prevalence of *stx* genes in stool samples (6.2%) was lower than the 9% previously reported by Kullin et al., 2015.^24^ Among the 53 isolates that formed mauve colonies on CHROMagar^TM^STEC, five were STEC (two serotype O26 and others non-typeable), four were enteroaggregative *E. coli* (serotype O104), two were atypical EPEC (serotype O101 and non-typeable) and two were typical EPEC (serotype O55). Serotypes O26 and O104 are among the top six STEC serotypes globally.^25,26^ CHROMagar^TM^STEC is intended for STEC culture; however, we detected other diarrhoeic *E. coli* pathotypes using this medium. The other pathotypes detected might have been hybrid strains that lost the *stx* genes or hybrid strains whose *stx* genes could not be detected using the primers employed in this study. Notably, bacteriophages carrying the *stx* genes are very quickly lost both in vivo and in vitro,^27^ and not all *stx* primers can detect all the *stx* gene variants.^28^

### Limitations

Not all STEC are tellurite resistant and may have been missed on CHROMagar^TM^STEC culture. This study only focused on the strains that formed mauve colonies. The stool samples in this study were collected between September 2014 and May 2015, and therefore the results of this study as regards prevalence of STEC may not reflect the entire year or areas of South Africa other than Cape Town.

### Conclusions

The in-house developed real-time PCR assay is a sensitive and specific alternative to the currently used diagnostic strategy. Due to the high false positivity rate, CHROMagar^TM^STEC can only be used as an adjunt to a more sensitive and specific assay such as real-time PCR.

## Trustworthiness

To the best of our knowledge, the findings of this study can be used as per the scope of the study and in light of the study limitations as clearly pointed out.

### Reliability

We confirm that the experiments conducted in this study will yield the same results during repeated trials using the same reagents and detection platforms.

### Validity

To the best of our knowledge, the findings of this study, as obtained using the methods we employed, are valid for the study area and season. The in-house developed real-time PCR may, however, be adopted in other laboratories in developing countries.
